# Reproductive toxicology: keeping up with our changing world

**DOI:** 10.3389/ftox.2024.1456687

**Published:** 2024-10-11

**Authors:** Laura B. Miller, Morgan B. Feuz, Ralph G. Meyer, Mirella L. Meyer-Ficca

**Affiliations:** Department of Veterinary, Clinical and Life Sciences, College of Veterinary Medicine, Utah State University, Logan, UT, United States

**Keywords:** male reproduction, infertility, aging, obesity, reproductive toxicology, endocrine disrupting chemicals, intergenerational, nicotinamide adenine dinucleotide

## Abstract

Reproductive toxicology testing is essential to safeguard public health of current and future generations. Traditional toxicological testing of male reproduction has focused on evaluating substances for acute toxicity to the reproductive system, with fertility assessment as a main endpoint and infertility a main adverse outcome. Newer studies in the last few decades have significantly widened our understanding of what represents an adverse event in reproductive toxicology, and thus changed our perspective of what constitutes a reproductive toxicant, such as endocrine disrupting chemicals that affect fertility and offspring health in an intergenerational manner. Besides infertility or congenital abnormalities, adverse outcomes can present as increased likelihood for various health problems in offspring, including metabolic syndrome, neurodevelopmental problems like autism and increased cancer predisposition, among others. To enable toxicologic studies to accurately represent the population, toxicologic testing designs need to model changing population characteristics and exposure circumstances. Current trends of increasing importance in human reproduction include increased paternal age, with an associated decline of nicotinamide adenine dinucleotide (NAD), and a higher prevalence of obesity, both of which are factors that toxicological testing study design should account for. In this perspective article, we highlighted some limitations of standard testing protocols, the need for expanding the assessed reproductive endpoint by including genetic and epigenetic sperm parameters, and the potential of recent developments, including mixture testing, novel animal models, *in vitro* systems like organoids, multigenerational testing protocols, as well as *in silico* modelling, machine learning and artificial intelligence.

## 1 Introduction

Toxic effects of substances that humans are exposed to in the form of environmental chemicals, drugs, foods, or foods additives, pose a significant risk to public health. In addition to toxic effects that directly affect an individual’s overall (somatic) health, adverse consequences for fertility and offspring health are a significant part of this health threat. In light of worldwide rising infertility rates (Aitken, 2024a; De et al., 2024), it seems essential to efficiently determine reproductive safety profiles of the large number of compounds that individuals and their environment are exposed to. About 30%–50% of infertility cases are due to male infertility. A complex interplay of environmental and external factors, like involuntary exposure to gonadotoxic agents and increasing age, combined with lifestyle factors, such as recreational exposures (e.g., to tobacco, alcohol, or recreational drugs) and obesity contribute significantly to the observed male fertility defects (Eisenberg et al., 2023; Eisenberg, 2022). Among the exposures implicated in rising infertility and sperm pathologies are endocrine disrupting chemicals (EDCs, e.g., phthalates, bisphenol A and various pesticides), heavy metals (lead, cadmium, mercury), and persistent organic pollutants like polychlorinated biphenyl and dioxins (Grazia Mele et al., 2024; Wdowiak et al., 2024; Ramsay et al., 2023), as well as climate change (Daniels and Berger Eberhardt, 2024).

Because of the potential severe impacts of toxic exposures, including to reproductive toxins and toxicants, on public health, many countries have tasked specific government agencies with protecting their populations by providing protective regulations and guidelines for appropriate toxicological study design. In Europe, the primary regulatory document is the European Union’s REACH Regulation (Registration, Evaluation, Authorization, and Restriction of Chemicals), of which regulation number 1907/2006 (Annex VIII-X, Points 8.7.2-4) focuses on reproductive toxicology testing (European Parliament and Council of the European Union). In the United States of America (United States), testing of reproductive toxicity is controlled by several agencies, including the Environmental Protection Agency (EPA) (U.S. Environmental Protection Agency, 1996), Food and Drug Administration (FDA), Occupational Safety and Health Administration (OSHA) and Consumer Product Safety Commission (CPSC), depending on the nature, intended use and exposure circumstances of the compound of interest, such as environmental chemicals like pesticides, or substances intended for human consumption like pharmaceuticals, cosmetics, or foods. An example for such guidelines is ICH S5 (R3) (Detection of Reproductive and Developmental Toxicity for Human Pharmaceuticals, which was developed by the International Council for Harmonization of Technical Requirements for Pharmaceuticals for Human Use (ICH) and is followed by the FDA (ICH). Similar guidelines are used by respective regulatory agencies in Japan, Korea, and China. Further guidance for study design and methodologies is found in the FDA’s so-called Redbook, a comprehensive guide for safety assessments of foods, with chapters IV.C (Sections 8, 9) for developmental and reproductive toxicity studies (Redbook, 2000). European regulations controlled by REACH use study designs according to OECD Test Guidelines 414, 421, 422.2, which have been widely adopted for American testing as well. The EPA further provides regulations OPPTS 870.3800 and 870.3700 for Reproduction, Fertility and Prenatal Developmental Toxicity Studies.

A common goal in toxicological testing is to determine exposure doses or concentrations of toxic agents that cause no adverse effects (No Observed Effect Level, NOEL) or the lowest dose or concentration with a statistically significant adverse observed effect (Lowest Observed Effect Level). Such metrics are useful to determine exposure thresholds that avoid direct toxicity. Their usefulness for safeguarding the health of future generations without direct toxic exposure, e.g., in an intergenerational or transgenerational manner, is unclear at this point. Additional in-depth evaluation of health and fertility of offspring in a multi-generational manner would be required to confirm or adjust those metrics.

Currently, most study design guidelines, including the OECD and the Redbook, recommend using rats (*Rattus norvegicus*) as test species, and less commonly mice (EPA OPPTS 870.3800). Depending on study objective and guideline, studies should use 10–24 young mature rats (aged 8–10 weeks) per sex and treatment dose. Guidelines for reproduction/developmental toxicity screening tests and combined repeated dose toxicity studies recommend a minimum of 10 young rats per sex and dose (OECD 421, 422, respectively). Extended one-generation reproductive toxicity studies and two-generation studies should use a minimum of 20 animals per sex, dose, and generation (OECD 443, 416). For males, effects on testis, spermatogenesis, sperm quality and quantity, mating behavior, fertility, and offspring development are evaluated.

It has been generally agreed upon that, to best protect human health, reproductive toxicology testing methods should continuously be adjusted to closely reflect current societies, living conditions and exposure circumstances. Current regulations are generally open to using study conditions that most closely resemble intended use and exposure circumstances of the to-be-tested chemical. For example, dosing regimens and modes of administration can be adjusted to closely follow intended use, or the most likely exposure circumstances, and modified protocols and (genetically modified) animal models of relevant disease phenotypes are permitted.

In this perspective article, we would like to draw attention to some current trends that affect human reproduction, including the increase in age, obesity, and exposure to environmental toxicants, that should be considered and addressed as important cofounding factors when designing adequate and timely reproductive toxicology testing protocols. Such factors include, for example, the human trend to delay reproduction to later points in life, along with the growing awareness of a role of increased paternal age and long-term environmental exposures in neurological and late-onset diseases in future generations (Feuz et al., 2024).

Furthermore, unlike animal models in tightly controlled scientific studies, humans are generally exposed to combinations of many chemicals with potential additive and synergistic effects (Kortenkamp et al., 2007), which has resulted in new concepts for toxicologic testing, such as testing effects of toxic agent mixtures, which are more representative of certain facets of the “exposome” than individual substances alone. Similarly, there are ongoing efforts to rethink and redesign current toxicity testing protocols to adequate test compounds with hormone-like, low-dose and non-monotonic effects, in particular EDCs (Schug et al., 2013).

Overall, evidence is increasing that human reproductive fitness is deteriorating due to a combination of changing public health and social parameters, like an increasing incidence of unhealthy body weights and delayed age to conception, together with chronic and simultaneous exposure to multiple environmental chemicals. Most of the current common testing protocols and model systems, while capable of detecting single potent reproductive toxic agents, might not be able to adequately assess the threat that cumulative and chronic low-dose exposure to multiple reproductive toxic agents pose to an increasingly sensitive population.

In the following sections, we point out some potential current challenges and limitations when using standard reproductive toxicology testing.

## 2 Current trends and concerns

### 2.1 Aging

While aging was once thought to be a general “wearing-out” process, current research suggests that aging is the result of a number of complex mechanisms that are influenced by both genetic and environmental factors (Sorrentino et al., 2014; Scieszka et al., 2023). Although still incompletely understood, the aging process is currently described by a set of defined molecular and cellular changes, the so-called hallmarks of aging, that include increasing genomic instability, telomere attrition, epigenetic alterations, loss of proteostasis, deregulated nutrient sensing, mitochondrial dysfunction, cellular senescence, stem cell exhaustion, disrupted intercellular communication, dysbiosis, disabled macroautophagy, and chronic inflammation (López-Otín et al., 2013; Schmauck-Medina et al., 2022). In addition, aging coincides with a decline in fertility and overall reproductive health (Carrageta et al., 2022). There is also a growing body of evidence indicating that advanced paternal age is associated with a deterioration in testicular function, decreased sperm quality, including reduced sperm concentration, motility, and morphology with increased chromosomal defects and DNA damage, as well as altered hormone levels (Feuz et al., 2024). This is relevant for men’s health, since normal testicular function and hormone production are not only essential for reproduction, but also for a healthy metabolism, longevity, and healthspan. Moreover, this also raises concerns about the health of children conceived by older men (Chan and Robaire, 2022).

Aging itself is an important factor in the assessment of many pharmacokinetic and pharmacodynamic properties of drugs and toxicants (Sorrentino et al., 2014; Jackson et al., 2017; Saghir et al., 2020). With increased age the activity of various detoxifying enzyme complexes declines, notably the cytochrome P450 enzymes, glutathione S-transferases and UDP-glucuronosyltransferases (Konstandi and Johnson, 2023; van Bezooijen, 1984). Together with the age-related decline in renal function (Papacocea et al., 2021), this results in a decreased ability of the body to metabolize and eliminate toxic agents, which allows an increase in concentration and bioaccumulation of a given toxic agent, and ultimately an increased effect of a given dose in aged individuals. Beyond effects of these mitochondrial and proteostatic dysfunctions, the cellular DNA repair capacity is lower in older individuals, which further exacerbates consequences of toxic insults. On the other hand, substances can have an effect on the physiological age of an individual, when they act as “gerontogens,” or age-promoting toxicants, which are environmental agents that can accelerate the aging process (Wise, 2022) and induce various cellular and molecular changes that are considered to be senescence markers (Wise, 2022; Muthamil et al., 2024; Moqri et al., 2024). Examples are telomere attrition, cell cycle arrest (e.g., by p21/WAF1 and p16/INK4), senescence-associated phenotype (e.g., TGF-β, interferons) and epigenetic changes in histone and DNA methylation, with the latter considered “aging clocks” with sensitivity to chemical exposures (Margiotti et al., 2023; Ashapkin et al., 2023).

It is well documented that maternal age is critical for reproductive success, and there is a general awareness that cumulative exposure to toxic agents, as expected to occur with increasing age, contributes to deteriorating oocyte quality, even though the underlying molecular mechanisms are still not well understood [e.g., reviewed by Dutta et al. (2022)]. Comparable information on the role of exposure to environmental toxins and toxicants in deteriorating male reproductive success with increasing paternal age is even more limited, particularly regarding the genetic and epigenetic health of male germ cells and, subsequently, the health of offspring from exposed men (Aitken, 2024a; Aitken, 2024b). Current toxicological testing protocols do not require an age-stratified testing design or, at a minimum, inclusion of age-specific models that could reflect older individuals, despite the trend of increasing paternal and maternal age in the population, and despite the fact that both are known to adversely affect reproductive success.

Overall, aging research, including reproductive aging, and the development of appropriate models for both basic research and toxicological studies has proven difficult due to our still limited understanding and entanglement of “aging” and “age-related diseases” or multi-morbidities, which lead to large individual variation (Bellantuono and Potter, 2016). Furthermore, aging phenotypes significantly vary across different species. Classic model organisms can only contribute limited information on human aging, so data from a combination of models are necessary for aging research (Holtze et al., 2021). The most straightforward method for modeling aging is using an old organism. However, research on naturally aged organisms is often expensive, time-consuming, and labor-intensive (Kõks et al., 2016; Cai et al., 2022). Therefore, aging models are often categorized as either natural aging models or accelerated aging models, with mice and rats being the most commonly used species due to their relative genetic similarity to humans, low housing costs, fast reproductive cycles, and short lifespans. Various animal models are often used for individual facets of testicular aging, as reviewed in Carrageta et al. (2022).

One important phenomenon that occurs during human aging is a decline in body-wide nicotinamide adenine dinucleotide (NAD) levels (Covarrubias et al., 2021). NAD, including its phosphorylated and reduced forms NADH, nicotinamide adenine dinucleotide phosphate (NADP), and NADPH, are central metabolites and enzymatic cofactors that are involved in numerous metabolic redox reactions, as well as in DNA repair and maintenance, epigenetic regulation, and autophagy. A sufficient pool of cellular NAD/NADH and NADP/NADPH is required for normal mitochondrial activity and reactive oxygen species (ROS) detoxification via glutathione activity (Srivastava, 2016). Also, in the mitochondrial compartment, NAD is utilized for adenosine triphosphate (ATP) production at multiple steps in the tricarboxylic acid (TCA) cycle and NADPH serves as a cofactor for cytochrome P450 enzymes that function to detoxify xenobiotics. Therefore, low NAD levels, as observed with aging, can lead to metabolic dysfunction and impaired mitochondrial detoxification reactions. Recently, our group developed a transgenic mouse model of Acquired Niacin-Dependency (ANDY), where removal of niacin from the diet results in body-wide decreases of NAD levels in young mice, which also results in a disruption of testicular function and spermatogenesis similar to that observed in old mice (Meyer-Ficca et al., 2022; Palzer et al., 2018). Using models such as the ANDY mouse may therefore be beneficial for toxicity testing that takes individual age and body condition into consideration.

### 2.2 Obesity

Another important current trend in Western societies is an increasing prevalence of obesity, with adverse effects for reproductive success in both women and men (Venigalla et al., 2023; Cannarella et al., 2024; Schon et al., 2024). Like aging, obesity has a significant impact on various physiological and metabolic processes, potentially resulting in altered pharmacokinetics and pharmacodynamics of xenobiotics (Risher et al., 2010; Tan et al., 2015; Apovian et al., 2023), and is thus expected to change sensitivity towards reproductive toxic agents. For example, in a recent report by Nie et al. (2022), single-cell transcriptomic analysis on testicular tissue from both young and old male donors demonstrated evidence for an age-related dysregulation in spermatogenesis. Here, however, the aging-effects appeared to be exacerbated in older men that were also obese compared to their age-matched non-obese counterparts.

In this context, it should particularly be studied how increased age and/or obesity influences sensitivity to EDCs, which have been identified as major threats to reproductive public health. Similar to “gerontogens,” “obesogens” are chemicals, including EDCs, such as tributyltin, bisphenol A, phthalates, among others, that are known to interfere with metabolic processes and promote obesity through several mechanisms in humans and animals (Amato et al., 2021; Darbre, 2017; Gupta et al., 2020; Amon et al., 2024). Obesogens can directly increase the number and/or size of adipocytes, cause a disruption in adipocyte function, as well as indirectly disrupt metabolic pathways, appetite control, microbiome composition, and increase calorie storage. On a molecular level, obesogen EDCs are hypothesized to function, at least in part, via interactions with ligand-mediated transcription factors that interact with nuclear hormone and/or steroid receptors to ultimately elicit changes in gene expression (Amato et al., 2021; Darbre, 2017). For example, peroxisome proliferator gamma (PPARγ) is a nuclear receptor highly involved in adipogenesis and is a major target investigated when evaluating the obesogenic potential of a given chemical. Activation of PPARγ requires heterodimer formation with retinoid X receptor (RXR) to bind to its target DNA sequence, which, in turn, increases transcription of adipogenic genes that stimulate adipocyte differentiation.

Additionally, the negative effects of obesogens are of particular concern regarding potential transgenerational effects passed from parents to offspring and during neonatal development (Amato et al., 2021). Throughout critical developmental periods, a growing fetus is more susceptible to the influence of exogenous xenobiotics and its plastic nature responds to hormonal signaling pathways. Thus, neonatal EDC exposure may disrupt sensitive systems and increase the predisposition for obesity later in life. However, the mechanism of obesogen action across generations is less clear. Epigenetic modifications and alterations in chromatin organization are thought to play a role, but more research is required in this area. In summary, aging and obesity are factors that alter susceptibility of individuals to toxic effects of xenobiotic substances because they change the metabolism of those agents. In addition, increased adiposity and age should also increase the effects and severity of long-term exposure to these compounds.

### 2.3 EDC exposure

EDCs are exogenous compounds of natural or synthetic origin that interfere with any aspect of normal hormone function, with adverse effects for exposed individuals and/or their progeny (Xin et al., 2015). Currently, more than 800 commercial chemicals are believed to interfere with the endocrine system. On a molecular level, EDCs amplify or attenuate hormone action through a variety of mechanisms (La Merrill et al., 2020). Among the recognized mechanisms are changes in hormone kinetics, e.g., altered hormone stability, transport into cells and transport and distribution within the body, direct interactions with hormone receptors with either activating or inhibiting effects, changed synthesis of hormones and/or hormone receptors, altered signal transduction and/or epigenetic modification in hormone-responsive cells. Altered epigenetic mark formation, e.g., persistently altered DNA methylation, chromatin modification or non-coding RNA expression, can further cause gene expression changes with long-term adverse consequences. EDCs exposure during critical time windows can affect development with long-term and even intergenerational consequences. An example is the observation that maternal bisphenol A exposure affected physiologic processes in offspring, with altered β-cell and mitochondrial function, gene expression and DNA methylation (Bansal et al., 2017). Several studies have shown that female exposure to EDCs may be associated with endometriosis, polycystic ovary syndrome, gynecologic tumors, and premature ovarian failure (Xu et al., 2021; Inman and Flaws, 2024), while exposure to EDCs in males can lead to a decrease in viable sperm due to impaired spermatogenesis or increase apoptosis, along with decreased motility, morphology and altered hormone levels (Sharma et al., 2020) [see Table 1; see (Walker et al., 2021) for a comprehensive overview]. Exposure to EDCs during fetal development can result in developmental abnormalities, including changed brain development (Cajachagua-Torres et al., 2024) and reproductive disorders. Further, if EDCs interfere with the epigenetic reprogramming of germ cells without preventing fertilization, adverse effects can persist across generations (Nilsson et al., 2023; Robaire et al., 2022), making inter/transgenerational studies crucial to evaluate long-term effects of parental exposure to EDCs. Indeed, in humans and in various animal models, parental EDC exposure was shown to decrease offspring viability and increase offspring reproductive dysfunction, behavioral abnormalities, and metabolic disorders (Zhang et al., 2024).

**TABLE 1:** Overview of select commonly encountered EDCs and their known effects on reproduction. More research is needed to confirm EDC function in the growing list of potential EDCs and to fully understand their effects on reproduction and development.

Endocrine disrupting chemical	Intended use and route of exposure	Effects in males	Effects in females
Bisphenol A (BPA)	Contaminated food, packaging	↓ sperm quality, ↓ motility (Wang et al., 2016), ↑ apoptotic germ cells (mouse) (Kalb et al., 2016)	↑ risk of miscarriage, ↓ oocyte yield, ↓ fertilization (Vessa et al., 2022)
De (2-ethylhexyl) phthalate (DEHP)	Medical devices, building and cleaning materials	↓ sperm production and viability, ↓ LH and FSH, seminiferous tubule atrophy (mouse) (Thacharodi et al., 2023), altered testosterone production (Jones et al., 2015)	↓ pregnancy rate, ↓ live birth rate, ↓oocyte yield (Hauser et al., 2016)
Methylparaben (MeP)	Cosmetics, food processing, toys, pharmaceuticals	More research needed	↓ fertility (Thomsen et al., 2016)
Glyphosate	Herbicide	↓ sperm count (rats) (Dai et al., 2016)	Possible reproductive system disease, more research needed (Kaboli Kafshgiri et al., 2022)
Dichloro-diphenyl-trichloroethane (DDT)	Contaminated water, air, soil, and food	Altered ratio of reproductive hormones (Bornman et al., 2018), ↓ sperm count, motility, and concentration (Toft, 2014)	↑ miscarriage rate (Jonsson et al., 1975) and altered menstrual cycles (Toft, 2014)
Dichloro-diphenyl-dichloroethylene (DDE)	Contaminated water, air, soil, and food	↓ sperm motility and mitochondrial function, and inhibits sperm capacitation (Tavares et al., 2015)	Lower quality embryos and ↓ fertilization (Petro et al., 2012)
Fenvalerate	Pesticide	↓ testosterone and sperm count, and ↑ germ cell destruction (Thacharodi et al., 2023), may inhibit progesterone production (Qu et al., 2008)	Cause structural changes in female genital organs, affect ovulation, cause degeneration of follicles and atrophy of endometrial glands (Marettova et al., 2017)
Polychlorinated biphenyls (PCB)	Industrial chemical	Change in reproductive hormones (Petersen et al., 2015) and ↓ sperm motility (Toft, 2014)	Altered menstrual cycles (Toft, 2014)
Vinclozolin	Fungicide	Testicular maldescent (rats) (Shono et al., 2004)	Virilization of females, affects progesterone and estrogen receptor expression (Buckley et al., 2006)
Dioxins	Bleaching, pesticides, combustion of medical plastic and waste	Altered breast development, ↑risk for mammary cancer (Diamanti-Kandarakis et al., 2009)	Possible endometriosis (Rier and Foster, 2003)
Genistein	Soy derived products	↓ sperm count, altered LH and testosterone levels (Rashid et al., 2022)	Altered menstrual cycle, ↓ ovarian function, and fertility (Jefferson et al., 2007)

### 2.4 Assisted reproduction

It is estimated that more than 10 million children have been born through assisted reproductive technology (ART) (Pinborg et al., 2023). Babies born from embryo transfer have an increased risk of altered birth weights, pre-term birth, and reduced growth rates, and babies from intracytoplasmic sperm injection (ICSI) may have increased risk of birth defects and decreased semen quality in male offspring (Berntsen et al., 2019). Despite sorting strategies to identify the healthiest sperm, selected sperm may still contain a high number of DNA strand breaks, abnormal methylation patterns, or altered non-coding RNA (ncRNA), all of which are (epi-)genetic contributors to fertilization and offspring health (Feuz et al., 2024; Alves et al., 2020). While the risks associated with ART are known, the underlying mechanisms are still being elucidated. For example, Sommer *et al.* found that polystyrene, a material commonly used for *in vitro* culture dishes, softens when in contact with aqueous media and then establishes a nanoscopic alkaline layer. This can trap ROS that naturally exude from the sperm that would be quickly neutralized *in vivo* but not in the *in vitro* culture dish (Försterling et al., 2017). How often the negative effects observed in ART offspring are caused by low quality gametes or by exposure to adverse conditions and reagents such as culture media, plasticizers, increased ROS, cryopreservation, and additional exposure to potential environmental toxic agents while undergoing ART still needs to be determined.

## 3 Modeling exposures in reproductive toxicology

### 3.1 Animal models

**FIGURE 1:**
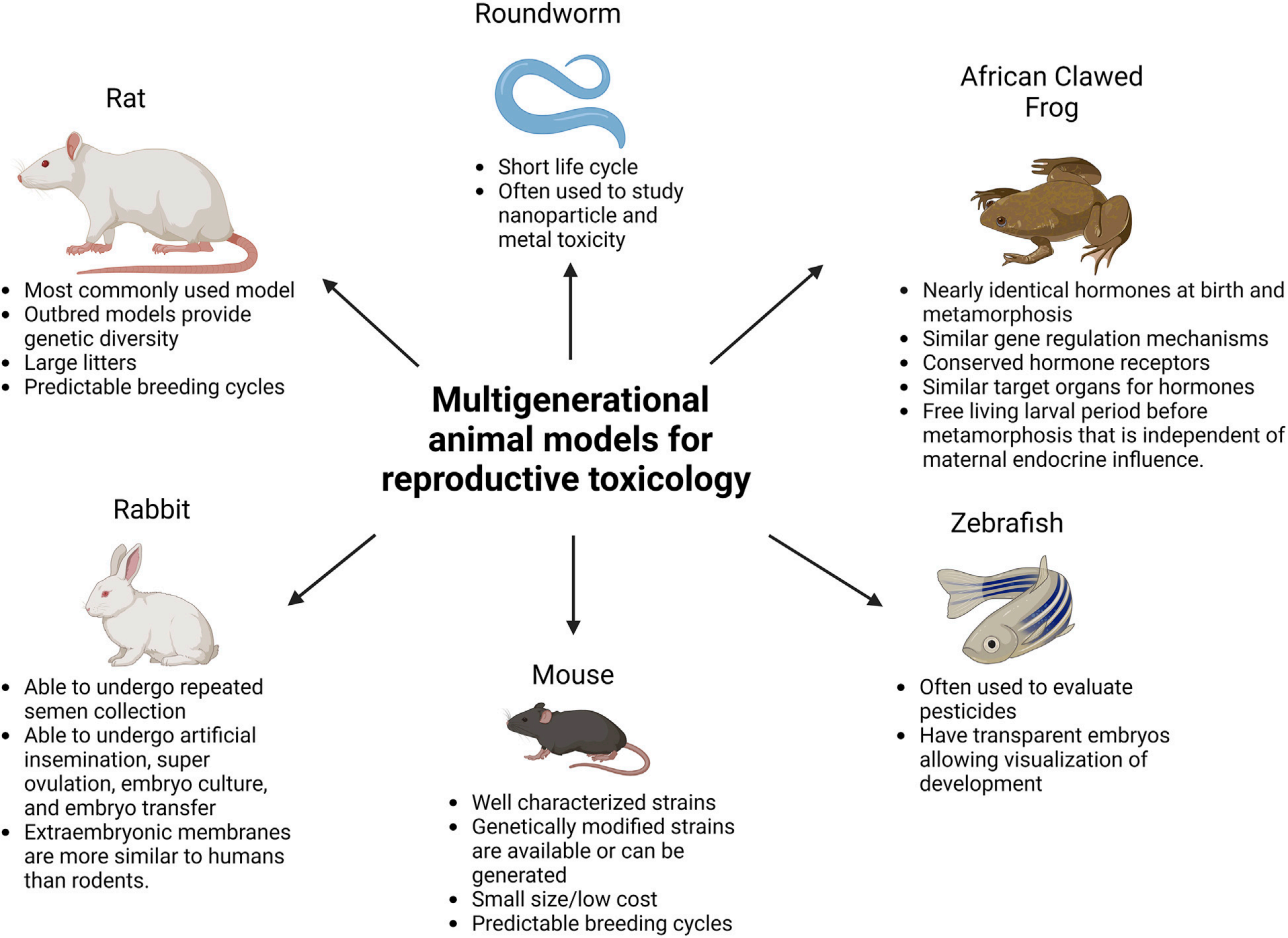
Commonly used multigenerational animal models for reproductive toxicology. Created with BioRender.com.

Reproductive toxicology research is not only needed to study the immediate effects of toxic agents on an individual, but also to determine the long-term consequences for their offsprings’ health. In order to identify current/future threats to safeguard the health of future generations, we need to understand better how environmentally induced alterations of the sperm epigenome occur, as well as the phenotypic changes they cause in the developing embryo. To assess these risks, multigenerational - including transgenerational and intergenerational - reproductive toxicology studies are needed. “Multigenerational” refers to effects seen in offspring whose mothers/fathers (F0) were exposed to a stimulus (e.g., stressful event, toxic exposure, etc.). Effects seen in offspring that were themselves exposed, either during fetal development or as germ cells, are considered intergenerational. Effects seen in F1 and F2 generations after maternal exposure, or effects in the F1 generation after paternal exposure fall into this category. Effects that persist in subsequent generations without direct exposure are considered transgenerational (Mbiydzenyuy et al., 2022). Such effects are thought to mostly be consequences of epigenetic changes in the germ line. In addition to traditional wild-type mice and rats (Zhang et al., 2024), genetically modified rodents (e.g., Takahashi et al., 2023) and other animal species are used to study multigenerational reproductive toxicology, including roundworms (Yang et al., 2016), zebrafish (Shen and Zuo, 2020), rabbits (Foote and Carney, 2000) and African clawed frogs (Buchholz, 2015). While each of these animal models provides unique benefits, it is important to keep in mind how these models differ from humans, including genetics, environment, and metabolism (see Figure 1).

### 3.2 Alternative and future model systems

Organoids are another alternative method to study reproductive toxicology in both males and females. An organoid is defined as a “3D structure grown from a single stem cell and consisting of organ-specific cell types that self-organizes through cell sorting and spatially restricted lineage commitment” (Cyr and Pinel, 2022). As many toxic exposures are known to impact development of reproductive organs, organoids provide a model to identify how toxic agents alter cell differentiation without using live animals. Organoids grown from human biopsies allow the study of human tissue responses to toxicants, as well as their impact on cell differentiation. While organoids have several advantages, they also have limitations. Organoids can mimic the physiology of a tissue, but they cannot replicate the entire organism. They also lack immune cells, neurons, and vascularity. Due to their lack of vascularization, diffusion of gases and nutrients may not penetrate to the center of the organoid leading to necrosis. This is considered one of the leading limiting factors or organoid use (Cyr and Pinel, 2022).

In recent years, *in silico* models have been created, which allow for high-throughput analysis of many toxicants and their potential effects on animal models. This is done by compilation of chemical structures and reliable toxicity reference databases that include data covering information on pesticides, drugs, cosmetics, and food additives. These models are able to quickly screen many potential toxins and toxicants and attempt to predict their effect, as well as help identify potential LOAEL values (Selvestrel et al., 2022). These rapid assessments of possible toxicant risks aim to assist in developing more effective *in vivo* study designs (Liu et al., 2022). While many of these models are being developed for general toxicology, several have also been developed to study reproductive toxicology as well (Liu et al., 2022; Kolesnyk et al., 2023; Zhang et al., 2017).

### 3.3 Modality of exposure

**FIGURE 2:**
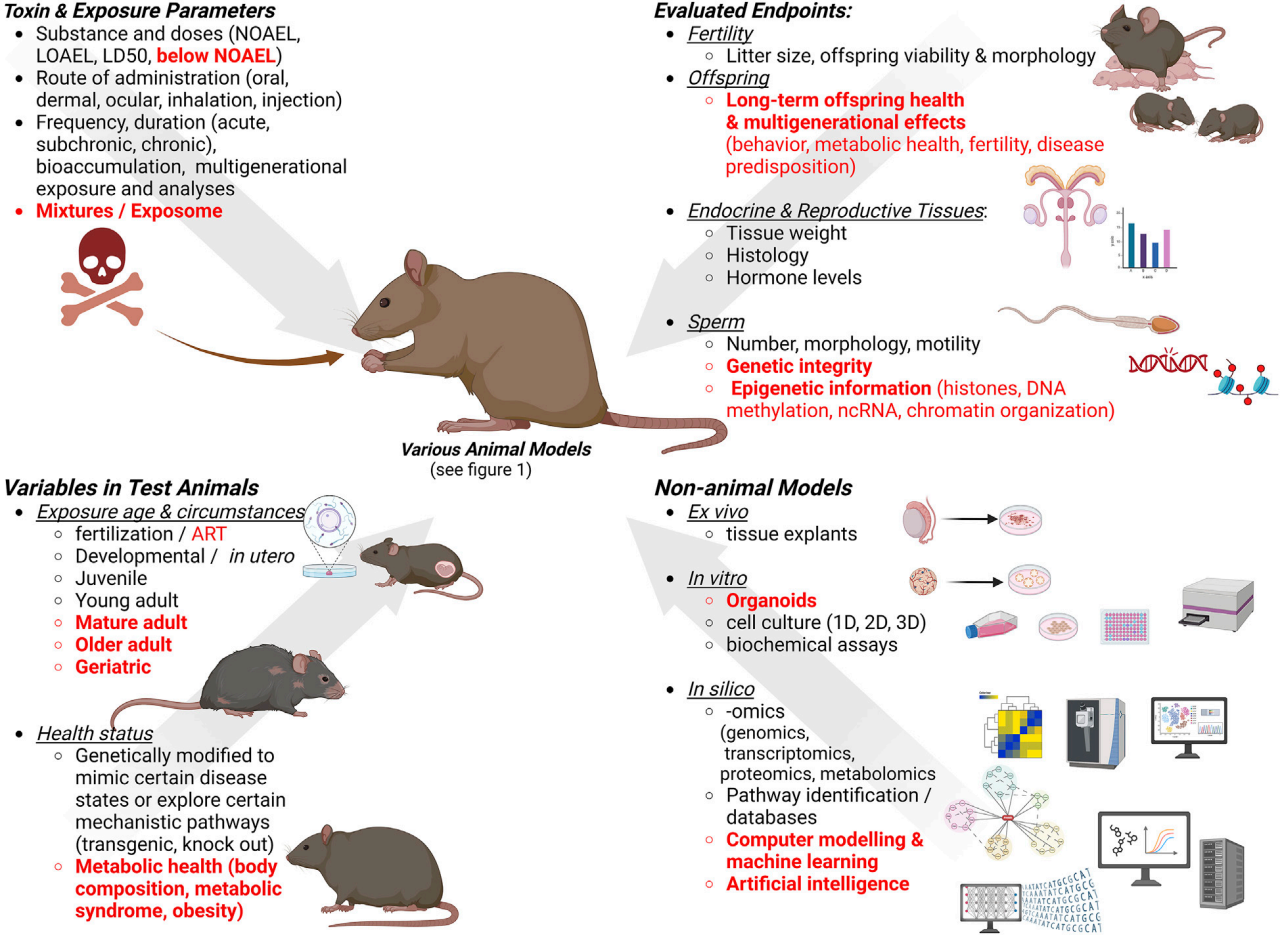
Considerations for designing studies for male reproductive toxicity testing. Gaps and new trends are indicated in red. Created with BioRender.com.

In addition to exposure routes that align with the chemical properties and bioavailability of a given toxin or toxicant to accurately reflect human exposure scenarios, reproductive toxicologic studies use different dosing regimens (details in Figure 2). Those include a single high exposure dose to identify immediate effects, repeated exposures that require a steady-state level of the toxic agent in the body, as in sub-chronic toxicity tests used to study the effects of multiple exposures to a given substance, and chronic toxicology studies requiring long-time exposure (Borgert et al., 2021). Sub-chronic studies usually have a 90-day duration, and chronic studies use repeated dosing over an animal’s lifespan. Depending on the specific chemical properties of a compound of interest, e.g., lipophilic substances and inorganic nanoparticles (Dantas et al., 2022), studies should test potential bioaccumulation and long-term toxicity as well. Further, single dose exposures are often administered during sensitive developmental time windows, e.g., during *in utero* development; however, they can also be administered to males or females prior to breeding. The *Developmental Origin of Health and Diseases* (DOHaD) concept and its expansion to *Parental Origin of Health and Disease* reinforces the importance of comprehensively evaluating sensitive time windows and long-term effects.

Toxic doses classified as “no-observed-adverse-effect level” (NOAEL) and “lowest-observed-adverse-effect level” (LOAEL) are used to establish repeated-dose toxicity (RDT) studies, with NOAEL being the highest experimental dose where no harmful response is observed, and LOAEL is the lowest dose when adverse effects appear compared to the control group. Currently, NOAELs are being used, in some instances, to calculate the margin of safety, reference dose, and acceptable daily intake (Selvestrel et al., 2022).

In addition, the Food Quality Protection Act is involved in assessing the risk that the use of multiple pesticides in food production might pose to consumers. Other chemical mixtures are not currently regulated, and most of the research has been focused on single chemicals. However, humans constantly encounter a variety of chemical mixtures including pesticides, household chemicals, cosmetics, food additives, among others. These chemicals are often introduced simultaneously and their combined effects, both on somatic and reproductive health, are currently understudied (Walker et al., 2021; Kortenkamp and Faust, 2018). How important adequate toxicologic evaluation of mixtures is for reproductive safety can be seen, for example, by a study from Conley et al. (2021), who observed male reproductive congenital defects after *in utero* exposure to a mixture of antiandrogenic chemicals at less than half of the NOAEL, delayed puberty after <1/4th of the mixture’s NOAEL, reduced testosterone production after <1/8th NOAEL, and significant gene expression changes at 1/16th NOAEL.

### 3.4 Study endpoints

Insights from toxicological tests depend on the evaluated endpoints. For male reproduction involving model organisms, the classical endpoints are hormone levels, weight of reproductive organs, sperm counts, morphology, motility, viability, as well as litter sizes and numbers (Hayes and Kobets, 2024). However, subtle changes to the sperm are not necessarily reflected in overt morphology or motility changes, yet still can have significant short- and long-term impacts on reproductive success and, importantly, long-term offspring health. More detailed sperm analyses should therefore be added to the battery of commonly assessed endpoints. Those include, but are not limited to, a more in-depth assessment of the sperm DNA genome and epigenome, for example, sperm DNA integrity, sperm chromatin compaction and composition, and histone and small nuclear RNA (snRNA) content of sperm (Kimmins et al., 2023). More subtle changes to offspring health, including behavior and cognitive functions, metabolic health, and cancer predisposition, should also be included in comprehensive toxicological evaluations, particularly given the ongoing rise in diseases like autism with a likely paternal origin, but currently not well-understood etiology. For example, Schrott et al. observed that exposure to air pollution was associated with sperm DNA methylation changes in loci with known relevance for neurodevelopment (Schrott et al., 2024).

## 4 Discussion

Arguably, effective reproductive toxicology testing should reflect living conditions in societies around the world that toxicology research and testing are intended to protect. It also needs to be continuously adjusted to changing societal trends. Currently, relevant trends in male reproduction are increasing paternal age, rising prevalence of obesity and increasing use of assisted reproductive techniques, as well as long-term and potentially synergistic exposures to many novel compounds and chemicals, including EDCs and nanoparticles. Further, test endpoints need to be expanded to include epigenetic and intergenerational health effects.

It is not feasible to comprehensively evaluate the toxicity profile of all environmental chemicals and potential mixtures in dedicated animal toxicity studies. Instead, identifying shared molecular initiating events (MIE), and resulting shared adverse outcome pathways (AOPs) for a group of similarly acting chemicals, are currently considered useful tools for assessing the toxic potential of substances, especially of EDCs, in an anticipatory “predictive toxicology” approach (Ankley et al., 2010). One AOP network investigating a large group of EDCs identified numerous shared key events contributing to their reproductive toxicology (Zilliacus et al., 2024). Currently, there are still major challenges to overcome (Svingen, 2022; Sewell et al., 2018). Besides the more commonly recognized problems, like non-linear effects, branching AOPs, and species differences, additional physiological parameters such as age, body condition and metabolic health, should be considered as factors in estimating potential toxic effects experienced by certain segments of a population. Further, integrated approaches (Hartung, 2009) to evaluate complex exposure scenarios and circumstances are needed (see Figure 2), and many challenges must be overcome to make “predictive and precision toxicology” feasible. While evidence for progressive detrimental decline of human reproductive health is rapidly mounting, it remains challenging to disentangle the extent to which individual lifestyle and environmental factors like exposure to toxic and endocrine disrupting chemicals, diet and age, contribute to this decline (Sharpe, 2024; Holmboe et al., 2024). The development of powerful AI systems and their recent utilization in toxicology seem promising steps to aid progress in reproductive toxicology (Kleinstreuer and Hartung, 2024; Hartung, 2023) and to augment classical approaches and to integrate “big data” generated in *in vitro* and animal testing.
